# A Handbook of Diseases of Women, Including Diseases of the Bladder and Urethra

**Published:** 1890-03

**Authors:** 


### A HANDBOOK OF DISEASES OF WOMEN, INCLUDING DISEASES OF THE BLADDER AND URETHRA.

BY DR. F. WINCKEL, PROFESSOR OF GYNÆCOLOGY, ETC., IN MUNICH, AUTHORIZED TRANSLATION, EDITED BY THEOPHILUS PARVIN, M. D., PHILADELPHIA, P. BLAKISTON, SON & CO., 1889.

Winzkel's Handbook of Diseases of Women has become a standard textbook in this country, and the only point which deserves notice in reviewing this second edition is to call attention to the very valuable addition which is made to the work by incorporating with it a condensed translation of the author's valuable monograph in diseases of the bladder and urethra. The work is thus rendered a complete treatise on the subject which it aims to present. As a matter of course, the views presented on many subjects are those held by our German conferers, and are therefore somewhat at variance with the accepted views in this country. There is also, as in all German works on the subject, an insufficient recognition of the work which has been done by American gynæcologists. The book is, however, no more faultless in this respect than might naturally have been expected. We can commend it to the practitioner as worthy of a place beside the treatises of Thomas, Emmet and Skene. For the student of medicine, we think that an American work is to be preferred to it.

